# The Nature and Treatment of Venereal Diseases

**Published:** 1874-09-15

**Authors:** 


*Received through Jansen, McClurg & Co.*

THE NATURE AND TREATMENT OF VENEREAL DISEASES. By Rob. A. Gunn, M.D. New York: Pp. 192.

The author of the work before us maintains, and in our estimation vainly attempts to prove, that all the various forms of venereal disease, as gonorrhœa, chancroid and syphilis, originates from one venereal poison. He claims that the virus is so modified by the condition of the patient at the time of his or her exposure, and by the length of time that elapses from the formation of the virus in one, before it is communicated to another, as to produce the three forms of venereal disease, with their various modifications.

We can but think that there is some mistake, or at least exaggeration, when the author tells us that he has known constitutional syphilis to follow gonorrhœa: even when a venereal sore could not be found after the closest examination.

In regard to his description of the various forms of venereal diseases, we have simply to say that it corresponds very closely to that of our older writers on this subject. In treatment, the local use of carbolic acid is very highly extolled. He assumes that tertiary symptoms seldom, if ever, appear if the acid is properly used in the primary local lesions, combined with constitutional treatment by tonics, and an intelligent use of the more mild alteratives, as podophyllin, ammoniæ chloridum, potas. iodid., etc. The tonics, however, he says, should have the precedence over the alteratives, the use of the alteratives being frequently suspended for several days. Mercurial medication is very strongly denounced, but the author's arguments to the effect that mercurial preparations have a tendency to produce various tertiary symptoms as lesions of the osseous system, fails to convert us to his opinion.

In justice to the author we must say that he writes in a clear, concise style, leading us with him from chapter to chapter, in a pleasant manner, notwithstanding that his and our opinions are frequently at variance.

